# Temporal evolution of tomographic findings of pulmonary infection in COVID-19

**DOI:** 10.31744/einstein_journal/2020CE5974

**Published:** 2020-10-02

**Authors:** Jose de Arimateia Batista Araujo-Filho, Marcio Valente Yamada Sawamura, Fernando Bin Teixeira, André Apanavicius, André Nathan Costa

**Affiliations:** 1 Hospital Sírio-Libanês São PauloSP Brazil Hospital Sírio-Libanês, São Paulo, SP, Brazil.; 2 Memorial Sloan-Kettering Cancer Center New York EUA Memorial Sloan-Kettering Cancer Center, New York, EUA.

Dear Editor,

We very enthusiastically read the excellent review article entitled “COVID-19 findings identified in chest computed tomography”, by Rosa et al.,^(1)^ published in the last issue of this journal. This manuscript shows the relevance of chest computed tomography (CT) in detecting the pulmonary manifestations of the coronavirus disease 2019 (COVID-19), presenting cases in a very didactic manner, but not discussing its role in the follow-up of the disease. Although we know this was not the scope of the authors, we would like to complement this discussion with some topics related to the impact of this imaging method in the prognosis and progression (long-term follow-up) of COVID-19.

We know that the early tomographic findings of pneumonia caused by the severe acute respiratory syndrome coronavirus 2 (SARS-CoV-2) are related to patterns relatively typical of involvement,^(2-4)^ which can provide important information for early diagnosis of and appropriate support to patients, particularly those with severe forms. However, it is not clear yet if the differences between the initial tomographic findings and those manifested in the course of the disease correlate with distinct clinical outcomes. In this context, some abnormalities usually suggestive of pulmonary fibrosis were described on chest CT of patients with COVID-19, such as parenchymal bands, linear and reticular opacities and architectural distortion,^(5)^ in addition to perilobular opacities suggestive of organizing pneumonia, mainly in the later stages of the disease (12 to 17 days after onset of symptoms).^(6)^ Although the prognostic relevance of these findings is still arguable, some authors suggested these tomographic signs could be associated to poorer prognosis.^(5)^ However, studies dedicated to the topic, with longer follow-up and including functional tests are required to clarify this issue.

Lessons learned from pathogens that cause other severe acute respiratory syndromes (SARS), including different types of coronavirus,^(7)^ taught that variable grades of pulmonary fibrosis were observed in subgroups of survivors, in the so-called pulmonary fibrosis after SARS. Nonetheless, the fibrosis after SARS differs from that occurring in interstitial lung diseases, since it does not have a progressive characteristic, although severe and limiting cases have been observed.^(8)^ Moreover, autopsies in fatal cases of COVID-19 have described the pathological findings of diffuse alveolar damage, fibrinous and organizing pneumonia, besides interstitial and extracellular matrix thickening, but with no mature pulmonary fibrosis documented so far.^(9)^ Therefore, it is assumed that at least some of the late findings on chest CT of patients with severe forms of the disease can be somehow related to organizing pneumonia secondary to viral infection.^(6)^ Considering that organizing pneumonia can potentially progress to pulmonary fibrosis, patients with suggestive or typical tomographic findings could benefit from long-term tomographic follow-up, and steroid therapy may be eventually considered.^(6)^

**Figure 1:**
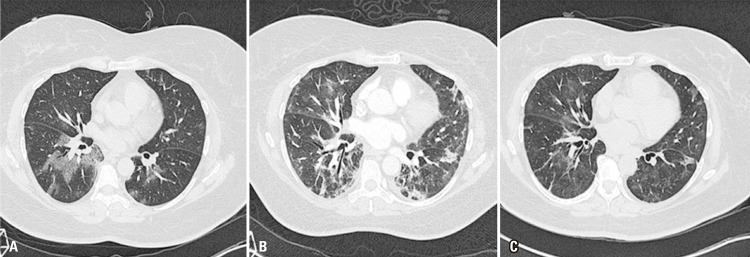
High-resolution CT scans showing progression of patient with severe pneumonia and COVID-19 (High resolution computed tomography – axial sections): (A) computed tomography at hospitalar admission, with bilateral patchy ground-glass opacities; (B) worsening of tomographic pattern at follow-up CT performed 17 days later, with emergence of parenchymal bands, reticular and some perilobular opacities, mild architectural distortion and irregular bronchial walls; (C) long-term CT follow-up (45 days after baseline CT) showing significant reduction of pulmonary abnormalities

To illustrate such discussion, we present a clinical case of a 56-year-old obese (body mass index - BMI of 33kg/m2), diabetic and hypertensive female patient, presenting dyspnea and cough for 5 days, with multifocal and bilateral ground-glass opacities (Figure 1A) at CT scan performed at hospitalar admission and COVID-19 infection confirmed by real-time polymerase chain reaction (RT-PCR). During the hospitalization she presented progressive hypoxic respiratory failure and was admitted, to the intensive care unit with severe acute respiratory syndrome (partial arterial pressure of oxygen/inspiratory oxygen fraction ratio - PaO_2_/FiO_2_ of 70). She was placed on mechanical ventilation support, and received anticoagulant, steroid (methylprednisolone 1mg/kg) and broad-spectrum antibiotic therapy. After improving symptoms of respiratory failure, and already on spontaneous breathing with nasal oxygen support, a new chest CT was performed (17th day of admission) and showed changes in the tomographic pattern, now presenting parenchymal bands, reticular and perilobular opacities, associated with mild architectural distortion, and irregular bronchial walls (Figure 1B). The patient progressed well, with gradual clinical improvement, and was discharged with dyspnea classified as Medical Research Council (MRC) 1, and oxygen saturation (SpO_2_) of 93% in room air. The late follow-up CT after hospital discharge (45 days after first CT) showed significant reduction of pulmonary opacities, architectural distortion and bronchial irregularities, suggesting inflammatory etiology and potentially reversible nature of such abnormalities (Figure 1C).

The temporal evolution of the reported case suggests that imaging follow-up may be useful to assess severity of pulmonary involvement in selected patients with severe forms of pneumonia caused by SARS-CoV-2, especially considering the difficulty to perform functional tests in these patients during the pandemic. We emphasize that some imaging findings that may potentially be considered sequelae in the course of the disease can have an inflammatory and reversible nature, as illustrated in the case presented. However, the literature has no reported yet an uniform or regular pattern of involvement that can guide the frequency of follow-up of these patients. While there are no available studies with long-term imaging and functional follow-up, the indication for CT follow-up of these cases should be considered in a judicious and individualized manner. Lastly, individual clinical conditions, contraindications, and cumulative radiation exposure must be mandatorily considered in this decision.
