# Supplementary material for: Temporal evolution of tomographic findings of pulmonary infection in COVID-19
Source: Einstein (Sao Paulo). 2020 Oct 2;18:eCE5974. doi: 10.31744/einstein_journal/2020CE5974 (PMC7531897; doi:10.31744/einstein_journal/2020CE5974)
Supplement: Translation

# Translation: Evolução temporal dos achados tomográficos da infecção pulmonar na COVID-19

**Authors:** Jose de Arimateia Batista Araujo-Filho, Marcio Valente Yamada Sawamura, Fernando Bin Teixeira, André Apanavicius, André Nathan Costa

**Affiliations:** 1 Brasil Hospital Sírio-Libanês, São Paulo, SP, Brasil.; 2 EUA Memorial Sloan-Kettering Cancer Center, Nova Iorque, EUA.

Caro Editor,

Com muito entusiasmo, lemos o excelente artigo de revisão intitulado “Achados da COVID-19 identificados na tomografia computadorizada de tórax”, de autoria de Rosa et al.,^(1)^ publicado no último número desta revista. O referido trabalho ilustra com casos didaticamente apresentados a importância da tomografia computadorizada (TC) de tórax na detecção das manifestações pulmonares da doença pelo coronavírus 2019 (COVID-19), sem discutir seu papel no seguimento evolutivo da doença. Embora saibamos não ter sido este um escopo dos autores, gostaríamos de complementar essa discussão com alguns pontos referentes ao impacto desse método no prognóstico e na evolução (sobretudo a longo prazo) da COVID-19.

Sabemos que os achados tomográficos iniciais da pneumonia pelo coronavírus da síndrome respiratória aguda grave 2 (SARS-CoV-2) apresentam padrões razoavelmente típicos de acometimento,^(2-4)^ o que pode oferecer importantes informações para o diagnóstico precoce e o suporte adequado aos pacientes, sobretudo aqueles com formas severas. No entanto, não está claro ainda se as diferenças descritas entre os achados tomográficos iniciais e aqueles que se manifestam no decorrer da doença se correlacionam com os distintos desfechos clínicos observados. Nesse contexto, foram descritas na TC de tórax de pacientes com COVID-19 algumas alterações usualmente sugestivas de fibrose pulmonar, como bandas parenquimatosas, opacidades lineares e reticulares e distorção arquitetural,^(5)^ além de opacidades perilobulares sugestivas de pneumonia em organização, sobretudo nas fases mais tardias de acometimento (após 12 a 17 dias do início dos sintomas).^(6)^ Embora a relevância prognóstica desses achados seja ainda discutível, alguns autores sugeriram que tais sinais tomográficos poderiam se relacionar a um pior prognóstico.^(5)^ No entanto, estudos dedicados ao tema com maior tempo de seguimento e incluindo testes funcionais são ainda necessários para elucidar tal questão.

Lições aprendidas com patógenos causadores de outras síndromes respiratórias aguda graves (SARS), incluindo outros tipos de coronavírus,^(7)^ ensinam que graus variáveis de fibrose pulmonar foram observados em subgrupos de sobreviventes, na chamada fibrose pulmonar pós-SARS. Entretanto, a fibrose pós-SARS se diferencia daquela que ocorre nas doenças pulmonares intersticiais, por não ter característica progressiva, embora casos graves e limitantes sejam observados.^(8)^ Além disso, estudos de autópsia em casos fatais de COVID-19 descreveram achados patológicos de dano alveolar difuso, pneumonia em organização e pneumonia fibrinosa e organizante, além de espessamento intersticial e da matriz extracelular, mas sem fibrose pulmonar estabelecida documentada até o momento.^(9)^ Assim, supõe-se que ao menos parte dos achados tardios na TC de tórax de pacientes com formas graves da doença possa estar relacionada a algum grau de pneumonia em organização secundária à infecção viral.^(6)^ Considerando que a pneumonia em organização tem potencial de progredir para fibrose pulmonar, pacientes com achados tomográficos sugestivos ou típicos podem ser beneficiados com seguimento tomográfico a longo prazo e, eventualmente, a corticoterapia pode ser considerada.^(6)^

Para ilustrar tal discussão, apresentamos a TC de tórax na admissão hospitalar de uma paciente do sexo feminino, de 56 anos, obesa (índice de massa corporal - IMC 33kg/m^2^), diabética e hipertensa, apresentando dispneia e tosse há 5 dias, com opacidades em vidro multifocais e bilaterais (Figura 1A) e infecção por COVID-19 confirmada por reação em cadeia da polimerase em tempo real (RT-PCR). Evoluiu com insuficiência respiratória hipoxêmica progressiva, sendo admitida em unidade de tratamento intensivo com síndrome respiratória aguda grave (relação pressão parcial de oxigênio/fração inspirada de oxigênio – PaO_2_/FiO_2_ de 70). Recebeu suporte ventilatório mecânico e foi medicada com anticoagulante, corticosteroide (metilpredinisolona 1mg/kg) e antibioticoterapia de amplo espectro. Após melhora da insuficiência respiratória e já em respiração espontânea com suporte de oxigênio, nova TC de tórax (17º dia de internação) evidenciou mudança do padrão tomográfico, com surgimento de bandas parenquimatosas, opacidades reticulares e perilobulares, associadas à leve distorção arquitetural e irregularidade das paredes brônquicas (Figura 1B). A paciente manteve boa evolução, com melhora clínica progressiva, tendo recebido alta com dispneia classificada na escala *Medical Research Council* (MRC) 1 e saturação do oxigênio no sangue (SpO_2_) de 93% em ar ambiente. O controle tomográfico após alta hospitalar (45 dias após primeira TC) evidenciou redução significativa das opacidades pulmonares, da distorção arquitetural e das irregularidades brônquicas, sugerindo etiologia inflamatória e natureza potencialmente reversível de tais alterações (Figura 1C).

**Figura 1:** Evolução tomográfica de uma paciente com pneumonia grave e COVID-19 (Tomografia computadorizada de alta resolução – cortes axiais): (A) tomografia computadorizada de admissão, com discretas opacidades em vidro fosco bilaterais; (B) piora do padrão tomográfico na tomografia computadorizada de controle realizada 17 dias depois, com surgimento de bandas parenquimatosas, opacidades reticulares e algumas perilobulares, leve distorção arquitetural e irregularidade das paredes brônquicas; (C) seguimento tomográfico de longo prazo (45 dias após tomografia computadorizada de base) evidenciando redução significativa das alterações pulmonares

A evolução temporal do caso apresentado sugere que o acompanhamento tomográfico pode ser útil na avaliação da gravidade do acometimento pulmonar em pacientes selecionados com formas severas de pneumonia por SARS-CoV-2, sobretudo diante da dificuldade de realização de testes funcionais nesses pacientes durante a pandemia. Ressaltamos que algumas alterações tomográficas potencialmente consideradas sequelares no curso da doença podem ter natureza inflamatória e reversível, como ilustrado no caso apresentado. No entanto, não foi ainda descrito na literatura um padrão uniforme ou regular de acometimento que possa guiar a frequência e o intervalo de seguimento desses pacientes. Até que estejam disponíveis estudos com seguimento radiológico e funcional a longo prazo, a necessidade e a cronologia no acompanhamento tomográfico desses casos deverão ser consideradas de forma criteriosa e individualizada. Por fim, condições clínicas individuais, contraindicações ao exame e dose cumulativa de radiação empregada devem ser mandatoriamente consideradas nessa decisão.
